# Foreign Summary

**Published:** 1840-01-11

**Authors:** 


### FOREIGN SUMMARY.

### Velpeau's Clinical Lecture on Ophthalmia.

### No. XIV.

*Local treatment of acute iritis—Mercurial ointment—Belladonna—Various collyria—Puncture of the cornea, a dangerous expedient—Chronic iritis—Efficacy of issues—Synechia—Operation for the removal of this—Application of belladonna—Critical examination into and denial of the existence of catarrhal and various other specific forms of ophthalmia.*

The influence which local treatment exercises over iritis is certainly slight—a fact which the deep-seated situation of the inflamed organ at once explains; I cannot, however, agree with M. Sichel, in considering it to be decidedly prejudicial. In many instances I have seen the iritis itself benefited by the use of local remedies, and you are well aware that they are valuable therapeutic agents in the treatment of the inflammation of the cornea and of the conjunctiva, which nearly always accompanies inflammation of the iris.

Local remedies may be divided into two classes. The first class comprises those which act directly on the iris; the second those which only act on the membrane through the medium of the circulation. In the first class we find the various mercurial, opiate, and belladonna ointments, the action of which we will now examine.

Although mercurial ointment has enjoyed, and still enjoys great reputation, it has never in my hands proved a valuable remedy. Indeed, I have so often employed it without deriving any benefit whatever from it, that I cannot now place much confidence in its efficacy. You ought early to become familiarized with the idea that a multitude of remedies employed by every practitioner, exercises, in reality, little or no influence over the disease against which they are directed; nor is it difficult to account for the apparent contradiction. In iritis, for instance, after some substance or other, say mercurial ointment, has been applied to the temples during several days, the inflammation disappears. It is at once inferred that has given way to the application of the ointment. But in all probability some of the general measures which we have already detailed, such as general or local bleeding, have been simultaneously employed, and it then becomes difficult to decide whether the iritis has been cured by the mercurial ointment, or by the other remedies to which the medical practitioner has had recourse. We must also bear in mind the length of time the disease has already existed, when we wish to ascertain the value of a remedial agent which we have employed; because iritis, in this respect, similar to all other inflammatory affections, does not last for ever, and may disappear after a certain lapse of time, although no treatment whatever has been directed against it.

When iritis is accompanied by severe pain, and it is considered advisable to guard against the contraction of the pupil, ointments, containing opium and belladonna are indicated, the proportions of these ointments being one drachm of opium or belladonna to an ounce of hog's lard. Belladonna having a peculiar action on the pupil, has been much used in the treatment of iritis; we will therefore devote a few moments to the examination of its value as a therapeutic agent in this disease. In the first stage of the malady, while the inflammation is still acute, belladonna should not be employed, as, instead of diminishing, it increases the inflammation. The action it then exercises over the iris may be compared to that of a remedy which would oblige an inflamed muscle to contract, in order to cure the inflammation. But when the inflammation has in a great measure subsided, and we have to fear, as sequelæ, permanent adhesions between the radiated fibres of the iris, or between the iris and the adjacent organs, belladonna is extremely useful, and will often, if judiciously employed, cause the destruction of any slight adhesions which may have formed. Among the various preparations of belladonna which are employed, there are one or two which, in my opinion, ought never to be used unless the inflammation has entirely disappeared. I cannot, for instance, understand how some practitioners can recommend the concentrated solution of belladonna to be instilled into the eye when the resolution is not complete. This solution is, in reality, an extremely irritating preparation, as is plainly seen if a few drops are introduced between the eyelids of a person whose eye is not inflamed:—the eye immediately becoming red and painful. It is not, moreover, necessary to employ it when the inflammation has not entirely subsided, as the iris may be easily acted upon, either by frictions with belladonna ointment, or by giving some preparations of belladonna internally. The principal object we have in view in employing belladonna, is to produce alternate contraction and dilatation of the iris, in order thereby to destroy the adhesions that may exist. To accomplish this, certain precautions are necessary. Were the belladonna given every day, its action being slow and gradual, the adhesions would not be ruptured, but merely elongated. We are, therefore, much more likely to succeed if we give the belladonna every two or three days, by starts, as it were, so as to cause the pupil to dilate suddenly.

Among the remedial agents which constitute the second class of local remedies, there are very few of any value. The various collyria of lead, iron, &c. are of little or no use, unless there be at the same time conjunctivitis or superficial keratitis. When, indeed, we consider that the surface on which they are applied is separated from the iris by the entire thickness of the cornea, and by the aqueous humour, we cannot be surprised that they should exercise but little influence over the disease. As, however, collyria, containing laudanum and belladonna, sometimes appear beneficial, they may occasionally be employed. When they are resorted to, the eyelids should be bathed several times a day with the collyria, and if the inflammatory stage is passed, a few drops may be instilled into the eye.

When the various agents which I have enumerated do not appear to arrest the progress of the inflammation, there are other local remedies which may be tried, as they have been strongly recommended by some authors. I might name, for instance, the distilled water of the laurocerasus, in which cyanuret of mercury has been dissolved. This collyrium has been much used by M. Carron du Villards; but on perusing what he has said on the subject, I do not find that there is any reason to suppose its efficacy to be greater than that of the laudanum or belladonna collyrium. I do not mean to say that the remedy is a bad one, but that its efficacy has not yet been satisfactorily demonstrated.

Before I conclude these remarks on the treatment of acute iritis, I must say a few words respecting a much more energetic remedy than those we have just examined; I allude to puncture of the cornea. Many of you will probably be surprised to hear that such a measure should ever have been proposed against inflammation of the iris, yet not only has it been proposed, but also put into execution. Mr. Wardrop is, I believe, the inventor of this plan of treatment; but since he introduced it into practice, it must have been frequently resorted to, as M. Weller speaks of the puncture of the cornea as of an operation generally adopted in acute iritis by practitioners. Mr. M'Gregor has practised the operation at least five-and-twenty times. M. Carron du Villards has often performed it; and an Italian surgeon says that he has punctured the cornea fifteen times on the same patient. The puncture of the cornea having, therefore, been so frequently resorted to in the treatment of acute iritis, I cannot pass it over in silence, especially as it is a measure which must be either decidedly beneficial or extremely dangerous.

Those who advise the cornea to be punctured in iritis, contend that the puncture evacuating the fluids which distend the eye, alleviates the acute pain felt by the patient, and that when the membranes of the eye are no longer distended, the inflammation is soon resolved. But I have not yet met with facts calculated to prove that such is really the case, and reasoning alone is not sufficient to establish the efficacy of a remedy in the treatment of disease. I have several times punctured the cornea in cases of iritis which presented some special indication, such as accumulation of pus in the anterior chamber, and in every instance the malady has been aggravated. The authors who have extolled this plan of treatment do not say under what circumstances they think it proper to resort to it, but merely state that it is a valuable remedy in iritis. But this is not sufficient: when a peculiar method of treatment is recommended, the precise indication for its use ought to be distinctly laid down. Even from the statements of those who have spoken the most highly of the operation, it would appear that it is not always unattended with danger, as we find that both Mr. Wardrop and Mr. M'Gregor have seen it followed by the disorganization of the cornea. In my opinion the puncture of the cornea in acute iritis is not a remedy, but a dangerous operation, much more to be dreaded than the disease against which it is directed.

Such are the principal remedial measures to which we can have recourse in the treatment of acute iritis. When they are judiciously and energetically employed from the very commencement of the disease, it generally yields in from six to twelve days. But when the iritis is accompanied by some other deep-seated lesion, such as retinitis, choroiditis, or disease of the vitreous humour, the result of the best directed treatment is far from being favourable.

*Chronic Iritis.*

Chronic iritis, considered as a primitive disease, has hitherto been but little studied, nor can we be surprised that this should be the case when we remember that most of the symptoms which characterize the acute form of the disease are absent, and that consequently its existence must often pass unperceived. If, however, I may be guided by my own experience, I should be inclined to think that chronic iritis is, in reality, by no means an uncommon malady, and that several of those shades of amaurosis which are described under the name of *imperfect* amaurosis, may be refered to this affection.

As one of the modes of termination of acute inflammation of the iris, chronic iritis has been frequently observed, and the peculiar characters which it then presents are so manifest that it may be easily recognised. When one eye only is affected, the colour of the iris of the inflamed eye is not the same as that of the one which remains healthy. If the eye is exposed to the light it becomes moist and watery. A sensation of uneasiness scarcely amounting to pain is felt in the orbit, and the sight is more or less disordered. On the anterior surface of the iris spots of various colours may be perceived. The pupil is more or less contracted, and appears to have partly lost its usual mobility; indeed sometimes it remains perfectly immoveable, assuming every imaginable shape. Sometimes also adhesion takes place between the pupillary circumference of the iris and the anterior surface of the crystalline lens. The transparency of the humours of the eye is often more or less impaired. The presence of these symptoms can scarcely leave any doubt in our minds as to the existence of chronic iritis, especially if they exist as a consequence of acute inflammation.

The prognosis of chronic iritis is generally unfavourable, the functions of the eye being seldom entirely restored even in the most successful cases.

The remedial agents likely to prove beneficial in the treatment of this affection are the same as those which are employed in the acute form of the disease, modified, however, in such a manner as to suit the chronic state of the inflammation. Thus purgatives and external revulsives are more especially indicated, whilst blood-letting, both general and local, should only be resorted to occasionally, and that with moderation. Issues are often advantageous, but I do not place them at the nape of the neck —the region generally chosen—as the sub-occipital fossa is better adapted for the application of such a remedy, from the quantity of cellulo-filamentous tissue which it contains. By judiciously combining these measures we sometimes may succeed in subduing chronic iritis, but in the majority of cases the most wisely ordained treatment fails to effect a cure.

Synechia, both anterior and posterior, is a frequent symptom in chronic iritis. The remedies which have been recommended against this form of adhesion of the iris may be divided into two classes, the surgical and the pharmaceutical. Some practitioners have proposed to destroy the adhesions by means of a needle introduced into the posterior chamber, through the sclerotica in posterior synechia, and into the anterior chamber through the cornea in anterior synechia. The operation is by no means difficult to perform, it is true, but it exposes the healthy parts of the iris to be lacerated or separated from their attachments, and may give rise to serious inflammation of the other membranes of the eye. We must also bear in mind that even when the adhesions have been destroyed, they may be reproduced the following day. Prudence, therefore, forbids our adopting so uncertain a plan of treatment, especially as less dangerous measures will sometimes suffice. Thus, the administration of belladonna may, by causing the pupil to dilate, occasion the rupture of the adhesions. But as I expatiated at some length on the efficacy of belladonna given in cases of synechia, when I spoke of the treatment of acute iritis, I shall not say any thing further on the subject. You must not, however, forget that when these remedies are employed, the treatment must be conducted in such a manner as to cause abrupt and not gradual dilatation of the pupil. The following is the plan I generally adopt:—I dilute a few grains of the extract of belladonna in a tea-spoonful of water, and instil two or three drops into the eye night and morning. I then allow it to rest for two or three days, until the pupil has returned to its natural state, when I again instil the solution of belladonna. I must once more remind you that I never use the solution unless the eye is perfectly free from inflammation. If a certain degree of inflammation still exists, I have recourse to frictions round the orbits with belladonna ointment, or I give it internally. By basing my practice on these principles I have sometimes successfully treated cases of chronic iritis accompanied by synechia.

*Specific Ophthalmia.*

I have hitherto, in the course of these lectures, avoided employing the epithets which ophthalmologists generally join to the real name of the various inflammatory affections of the eye, as I wished first to make known to you my own opinions respecting this class of diseases. Now, however, that we understand each other—now that I have fully explained the peculiar views which I entertain—we are prepared to discuss the question of the existence or non-existence of specific ophthalmia.

Before I enter into the examination of this interesting question, I think it is necessary to state, once for all, that in what I am about to say I have not the slightest intention to be personal. I know too well that science has nothing to gain from personal quarrels to wish to engage in them. When, therefore, I mention names, which I shall occasionally be obliged to do, I wish it to be understood that I have merely in view the opinions professed by those to whom I allude. I consider it my duty to attack doctrines which I look upon as erroneous, even should I by so doing incur the displeasure of persons who may be rather too susceptible. Such, indeed, is the conduct which all true friends to the advancement of science ought to follow.

It is of much greater importance than is generally believed to determine whether the inflammatory affections of the eye are or are not of a specific nature. Ophthalmologists of the German school recognise a great number of specific forms of inflammation, the existence of which rest, in my opinion, on theoretical ideas, and not on an accurate and attentive study of nature. I feel myself called upon to do all in my power to prove the falsity of their views; the more so, as the elimination of so extensive a class of diseases would evidently tend much to simplify ophthalmology. I do not for a moment doubt that the state of the constitution, or a special affection, may exercise more or less influence over diseases of the eye, for this is a fact which is proved in the most satisfactory manner by daily experience; why, indeed, should the eye be shielded from such influence more than any other organ? I merely assert, convinced as I am by an immense number of facts of the truth of my opinions, that it is absolutely false to say that diseases of the eye assume peculiar anatomical characters because the patient is under the influence of a peculiar general affection, and that we may judge of the constitution of a person labouring under ophthalmia from the peculiar symptoms which that ophthalmia presents. Were this doctrine the expression of facts, and capable of demonstration, or, in other words, were not the different specific forms of ophthalmia which have been denominated *catarrhal*, *rheumatic*, *scrofulous*, *venous*, &c., mere creations of the imagination of those who describe them, we should necessarily meet with symptoms which would distinguish them from the simple inflammatory affections of the eye. But a conscientious and attentive examination of nature will show us that such is not in reality the case; indeed, I have frequently, as most of you must acknowledge, given, at the bedside of the patient, the most irrefragable evidence of the non-existence of these peculiar symptoms. I therefore feel authorized to give it as my decided opinion that the various specific forms of ophthalmia, about which so much has been said by the German school, and which are so strenuously defended by those who endeavour to propagate the ideas of Beer among us, do not exist, and ought not consequently to be admitted among the inflammatory diseases by which the eye may be attacked. I must, however, except from this general condemnation syphilitic ophthalmia, a class of diseases which may certainly be looked upon as specific. In order to convince you that I view in a proper light the question that is before us, we will attentively examine the symptoms which are attributed to each of these supposed specific affections.

It is scarcely necessary in the present state of science to allude to those forms of ophthalmia to which the epithets of *variolous*, *dartrous*, *hæmorrhoidal*, *menstrual*, *abdominal*, &c., have been given. You must all perceive how absurd, how ridiculous even, it would be to recognise them. Were we to do so we should also be obliged to recognise cardiac, intestinal, meningeal ophthalmia, indeed as many forms of inflammation of the eye as there are diseases. I intend, therefore, merely to speak of those specific affections on which the greatest stress is laid by my opponents, viz., *catarrhal*, *arthritic*, *rheumatic*, *scrofulous*, and *syphilitical* ophthalmia. That we may proceed with order in this discussion, we will first carefully examine the descriptions which authors give of these affections, and then, by establishing a parallel between the symptoms, which are attributed to them, and those which the simple ophthalmia offer, we shall easily discover on which side lies the truth. At the same time we must remember that for a morbid affection of a tissue to be separated from all other affections which may occur in that tissue; for it to be considered a special disease, something peculiar must necessarily be found in its symptoms, its progress, its treatment; in a word, it must have, as it were, a separate existence. Otherwise pathology would be an inextricable labyrinth.

M. Sichel being with us the representative of the doctrines which I now oppose, it is in his own work that I shall take the description of the symptoms which are supposed to characterize specific ophthalmia. This interesting and important question deserves much greater development than I shall now be able to give to it; yet, however brief I may be, I feel certain that what I am about to say will prove sufficient to convince those who listen to me without any feeling of prejudice, especially if they have followed my visits in the wards, and examined along with me the numerous cases we have received and treated this year.

*Catarrhal Ophthalmia.*

The seat of this affection is said to be the mucous membrane of the eye—the conjunctiva. The following are the symptoms by which it is supposed to be characterized:—At first the patient complains of slight itching of the eyes, especially of the inner canthus; the eyelids feel rather stiff, and a little mucus collects in the morning at the inner angle of the eye, and sometimes on the free edges of the palpebræ. At the same time the palpebral conjunctiva begins to present the catarrhal form of vascularization, that is, it appears of a rosy hue, and the vessels which it contains form parallel streaks, which pass from the free to the attached margin of the eyelids, and lose themselves in that portion of the conjunctiva which covers the sclerotica. Before we go any further, I must ask every attentive and impartial observer if he can discover in these symptoms any thing more than the first stage of mucous or glandular blepharitis; and if, on the other hand, the symptoms of the latter disease are modified when the patient is under the influence of a catarrhal affection? But, to continue. The vascular streaks which I have just mentioned, soon become more numerous; they are sometimes parallel, sometimes interlaced so as to form an irregular network. The entire palpebral conjunctiva becomes of a yellowish vermillion red colour; and the vascularization, extending to the ocular conjunctiva, assumes a peculiar aspect "pathognomonic of the catarrhal affection." The injected vessels are of a pale red colour; they are nearly parallel to one another, and, following a slightly tortuous direction as they pass on from the circumference of the conjunctiva towards the cornea, become gradually smaller until they terminate at the distance of a line or two from that membrane. The conjunctival redness is, therefore, always separated from the cornea by a white zone, which is formed by the portion of the mucous membrane that has remained healthy. There is neither photophobia nor epiphora. The secretion of mucus becoming gradually more abundant, it concretes during sleep on the free margin of the eyelids, and at the inner canthus, so as to form crusts, which the patients easily rub off in the morning. In the majority of cases the palpebral conjunctiva is covered with granulations of variable size. The pain which the patients feel is superficial, and is compared by them to that sensation that fine sand would produce were it interposed between the palpebræ and the eye. This symptom is generally more intense towards evening than at any other time, especially if the patient attempts to work by artificial light.

In what authors call the third stage of the disease, the injected conjunctival vessels are more numerous, of a larger size, and advance as far as the circumference of the cornea; then it is that we meet with all the varieties of chemosis.

Such are the principal symptoms which are attributed to "catarrhal ophthalmia." If you will take the trouble to compare them with those which I described when speaking of the various forms of blepharitis and conjunctivitis, you will at once see that the morbid phenomena are the same, and that the existence of these symptoms may be accounted for in the most satisfactory manner without its being necessary to have recourse to the supposition of a special affection. If any of you still entertain doubts respecting the identity of these symptoms, I would advise them to examine, with M. Sichel's book in their hands, several cases of blepharitis and conjunctivitis that we have now in our wards. They will find that whether the patient be labouring under a catarrhal affection or not, the same group of symptoms is present, and that the only modifications which they offer are those which are caused by the variable intensity of the inflammation.

